# Ethyl P-Methoxycinnamate: An Active Anti-Metastasis Agent and Chemosensitizer Targeting NFκB from *Kaempferia galanga* for Melanoma Cells

**DOI:** 10.3390/life12030337

**Published:** 2022-02-24

**Authors:** Subehan Lallo, Besse Hardianti, Sartini Sartini, Ismail Ismail, Dewi Laela, Yoshihiro Hayakawa

**Affiliations:** 1 Faculty of Pharmacy, Hasanuddin University, Makassar 90245, Indonesia; sardj@farmasi.unhas.ac.id (S.S.); ismail@unhas.ac.id (I.I.); dewiprimayanti@unhas.ac.id (D.L.); 2 Sekolah Tinggi Ilmu Farmasi Makassar, Makassar 90242, Indonesia; besse.hardianti@stifa.ac.id; 3 Institute of Natural Medicine, University of Toyama, Toyama 930-0194, Japan

**Keywords:** *Kampferia galanga*, ethyl p-methoxycinnamate (EPMC), p38α, phospho-Akt (Ser473), NFκB, B16F10-NFκB Luc2, chemosensitizer, anti-metastasis

## Abstract

The most common type of skin cancer is melanoma. While significant advances in chemotherapy have occurred in a few instances, only marginal progress has been made in treating metastatic melanoma. Natural medicine has traditionally been used to treat various illnesses, including cancer. The purpose of this study was to identify the active compound in *Kaempferia galanga*, which could be used to treat melanoma as an anti-metastasis and chemosensitizer agent. The active compound in *K. galanga* was isolated and identified using chromatography and spectroscopy techniques, and given six compounds. Inhibitory activity on NFκB activation and cell viability was determined using reporter assay methods. Among the isolated compounds, ethyl p-methoxycinnamate (EPMC) demonstrated potent NFκB inhibitory activity against melanoma cell B16F10- NFκB Luc2 with an IC50 of 88.7 μM. Further investigation was conducted by evaluating the anti-metastasis effect of EPMC in vitro by using wound-healing assays, invasion tests, and molecular mechanism assays using Western blotting. NFκB has been implicated in tumorigenesis through the PI3K/Akt/NFκB pathway. The results of this study indicated that EPMCs act as inhibitors of p38 and thereby Akt phosphorylation inhibitors at serine 473, inhibiting NFκB-dependent transcription. Further analysis with paclitaxel demonstrated that the combinations could sensitize to apoptosis in response to well-known chemotherapy agents. Additional studies were conducted using the human melanoma cancer cell line SK-Mel 28. Along with the induction of apoptosis, we observed an increase in p-γH2AX expression (a molecular marker for double strand breaks in DNA damage) in response to treatment with paclitaxel and EPMC. The result showed EPMC to be a potential, viable adjuvant for improving the clinical efficacy of anti-metastatic and cancer chemotherapy.

## 1. Introduction

Drug discoveries from natural medicine still receive attention from researchers. Some clinically used drugs have been discovered from natural medicine, proving the efficacy of these medicines. An investigation of drug candidates that were introduced into the market between 1981 and 2002 revealed that 28% were derived from natural products [1]. This implies that natural products are a valuable source of new drugs, as well as good lead compounds for further modifications during drug development.

Medicinal plants are the main source of drug discovery in traditional medicine. They have a secondary metabolite, which has been reported for its pharmacological activity. Almost all the plant family have been reported for their biological activity, including Zingiberaceae. An investigation of an isolated compound from *K. galanga* mainly reported it to contain essential oil [2]; however, it is widely known that plants collected from distinct altitude habitats possess distinct essential oil characteristics [3]. *Kaempferia galanga,* belonging to the Zingiberaceae family, is a plant that is widely used as a traditional medicine in Asia. According to the study by Khairullah at al., the plant is a stemless herb derived from tuberous rootstocks with a fibrous cylindrical root. It has dark reddish-brown skin, and the soft interior is nearly white [4]. It is empirically used as a single plant or as a complementary ingredient in a medicinal formula. This plant grows easily and thrives in tropical climates such as Southeast Asia. The rhizome of the plant is mostly used as a traditional medicine, either in its fresh or in dry form.

The empirical uses of the rhizome *K. galanga* were reported by Elshamy et al., as a cure for metabolic disorder, inflammation, urinary tract infection, fever, coughs, hypertension, erectile dysfunction, abdominal and gastrointestinal ailment, asthma, wounds, rheumatism, epilepsy, and skin diseases [5]. In Southeast Asian countries, this plant is used for the treatment of different kinds of diseases [6]. Various research results have proven that this plant has anti-inflammatory [7], anti-angiogenic [8], anti-cancer and anti-proliferative activities, among others [9]. Kumar et al. researched the chemical constituent of *K. galanga* rhizome, showing that this plant contains compounds such as esters, terpenoids, flavonoids, thiourea derivatives, polysaccharides, diarylheptanoids, phenolic acids, phenolic glycoside and cyclic lipodepsipeptide types [10]. Propanoic acid, pentadecane, and ethyl p-methoxycinnamate (EPMC) compounds were reported as the most abundant compounds from the essential oil of this plant [11].

Various testing methods have been developed to explore the potential of materials that can be used as drugs, especially for cancer. Several drugs were found and used but were not able to completely overcome the problem of the disease. Efforts were made to find cancer drugs that can inhibit NFκB activity and are not cytotoxic to normal cells. The NFκB gene plays a role in the development of cancer in humans. There have been important concerns about NFκB’s role in the initiation, development, metastatic, and treatment resistance of human cancer. The suppression of NFκB in myeloid cells or tumor cells usually leads to tumor regression, which makes the NFκB pathway a promising therapeutic target [12].

This study aimed to find medicinal ingredients sourced from traditional medicines, which have the ability to inhibit NFκB with low toxicity. The material’s ability to increase the sensitivity of drugs which have been used as cancer drugs to date was also tested.

## 2. Materials and Methods

### 2.1. Plant Extracts and Compounds

*K. galanga* was collected from the South Sulawesi Province, Indonesia. The dried rhizomes of plants were extracted with 70% ethanol using the maceration method. Liquid extracts were then evaporated and lyophilized to obtain an EtOH extract [13]. Further separation was performed by combination column chromatography and preparative thin layer chromatography (PTLC) to give 6 isolated compounds. Purification of the isolated compounds was carried out with PLTC silica gel and n-hexane:EtoAc (7:3) as a mobile phase to give 6 pure compounds. The structure of the isolated compounds was determined using NMR and MS spectroscopy with a comparison to the references. Paclitaxel was purchased from Fuji film Wako Pure Chemical Co., Osaka, Japan, SKN 5312.

### 2.2. Cells and Reagents

B16F10 and SK-Mel 28 cell lines were obtained from the American Type Culture Collection and maintained at 37 °C in Eagle’s minimal essential medium (Nissui Pharmaceutical Co., Ltd., Tokyo, Japan) containing 10% fetal bovine serum (FBS; Nichirei Biosciences, Inc., Tokyo, Japan). In order to create an NFκB-mediated luciferase gene expressing B16F10 cells (B16F10 NFB), B16F10 cells (5 × 10^5^/well) were seeded in a 6-well plate and the pGL4.32 vector was transfected with Lipofectamine 2000. The cells were cloned using limiting dilution after being selected with Hygromycin B (200 g/mL). B16F10 NFκB transfectants, or B16F10 CMV control cells (1 × 10^5^/well), were cultured in a 96-well plate and treated with TNF (0.1–100 ng/mL) to assess the NFκB response in vitro. After 6 h of incubation, luciferase activity was measured using a multiplate reader (2030 ARVO X; Perkin Elmer Life Sciences, Boston, MA, USA) [14].

### 2.3. Cell Viability

B16F10-NFκB-Luc2 cells were plated at a final concentration of 2 × 10^4^ cells/well in a 96-well plate. After 24 h of incubation, the cells were pretreated with 50 μg/mL of extract or compounds and further incubated for 24 h. Subsequently, 10 µL of WST-8 (FUJIFILM Wako Pure Chemical Corporation) solution was added to the cells, which were incubated for an additional 1 h in a humidified atmosphere (37 °C and 5% CO_2_) to allow for the formation of formazan dye and to increase sensitivity. Then, the absorbance was measured with a microplate reader (Sunrise™; Tecan Group Ltd., Männedorf, Switzerland) at wavelengths of 450/620 nm. Cell viability was determined based on the absorbance of the soluble formazan dye generated by the living cells. The same method was followed to determine the viability of B16F10-NFκB-luc2 cells treated with selected extracts in a concentration-dependent manner. The WST-8 solution was added and used according to the manufacturer’s instructions. The viability of the treated cells was calculated as a percentage [13,14].

### 2.4. NFκB Reporter Gene Assay

B16F10-NFκB-Luc2 cells were maintained in EMEM supplemented with 10% FBS, 1 mM L-glutamine, and antibiotics (100 U/L penicillin and 100 mg/L streptomycins) at 37 °C in a humidified atmosphere containing 95% air and 5% CO_2_. The cells were seeded in black 96-well plates at a density of 2 × 10^4^ cells per well and then incubated for 24 h. Subsequently, they were treated with the extracts, and an equal concentration of the solvent vehicle was included as a control after 24 h. At the end of the assay, 900 µg/mL D-luciferin was added, and the plates were incubated for another 30 min. Luciferase activity was measured according to the luminescence of firefly luciferase, which was quantified using IVIS LUMINA II and Living Image 4.2 software (Caliper Life Science, Waltham, MA, USA) by determining the light emitted from cells. Data were expressed as photons/s and the fold NFκB activity was calculated as a total flux of extracts (photons/s) is divided by a total flux of vehicle as control (photons/s) [13,14].

### 2.5. Wound Healing Assay

B16F10G5-Luc cells were seeded at a concentration of 1 × 10^5^ cells/well in a 24-well plate and allowed to form a confluent monolayer for 24 h. After scratching the monolayer with a sterile pipette tip (1 mL), it was washed with medium to remove floating and detached cells and photographed (time 0 h). For 24 h, these cells were treated in medium containing 50 μM concentrations of EPMC. Scratched areas were photographed (magnification ×10 using a Biozero BZ-8000 microscope, Osaka, Japan) at 0 h as T-0 and again 24 h later as T-24 to assess wound healing. The wound closure percentage was calculated as follows: wound closure percent = 1 (wound area at T-24/wound area at T-0) 100 percent, where T-24 is the time after wounding and T-0 is the time immediately after wounding [15,16].

### 2.6. Transwell Invasion Assay

A transwell chamber (Costar No. 3422, Cambridge, MA, USA) with an 8.0 µm pore size polycarbonate filter (Whatman, Clifton, NJ, USA) on the lower part and 1 g of Matrigel on the upper part of the filter was pre-coated with 10 g fibronectin (Fujifilm Wako Pure Chemical Corporation, Tokyo, Japan). B16F10 G5-Luc cells were pre-treated with 50 μM EPMC overnight before being suspended in serum-free media in the upper transwell chamber (3 × 10^5^ cells/100 μL/chamber) and placed in a 24-well plate (lower compartment of chamber) with media containing 0.1 percent (*v*/*v*) BSA, then incubated at 37 °C for 7 h. Following incubation, the invading cells on the membrane’s lower surface were fixed in methanol, stained with hematoxylin and eosin, and wiped with cotton swabs to remove non-invasive cells. The cells that invaded through the filter were photographed at magnification ×10 using a Biozero BZ-8000 microscope (Keyence, Osaka, Japan). Invading cells on the membrane were randomly selected in five visual fields, and the average value in the five fields was used to calculate the migration index. Image J software was used to count the cells that had infiltrated the filter [17].

### 2.7. Western Blotting

B16F10 G5-Luc Cells (10^6^ cells/well) were treated for 12 h with the tested compounds EPMC 50 μM and PTX 20 μM. Trypsinized cells were harvested by adding PBS and centrifuged for 10 min at 14,000 rpm and 4 °C. The supernatant was then discarded, and the cells were lysed in a whole-cell lysis buffer (25 mmol/L HEPES, pH 7.7, 300 mmol/L NaCl, 1.5 mmol/L MgCl_2_, 0.2 mmol/L EDTA, 0.1 percent Triton X-100, 20 mM *β*-glycerophosphate, 1 mM sodium orthovanadate, 1 mM phenyl-methylsulfonyl fluoride, 1 mM dithiothreitol, 10 mg/mL of aprotinin, and 10 mg/mL of leupeptin). Cell lysates were electrophoretically separated using 10% SDS-PAGE and electrophoretically transferred to Immobilon-P nylon membranes (Millipore, Bedford, MA, USA). The membranes were blocked for at least 2 h with Block Ace (Dainippon Pharmaceutical, Co. Ltd., Osaka, Japan) before being probed with the indicated primary antibodies at 4 °C overnight, followed by incubation with horseradish-peroxidase-conjugated secondary antibodies (1:1000 dilutions) at room temperature. ECL reagents were used to visualize the bands (Amersham Bioscience, Piscataway, NJ, USA). Primary antibodies used were specific to p65 (LF6, #6956), p-p65 (93H1, #3033) (Cell Signaling Technology, Beverly, MA, USA), β-actin (C4, sc-47778) (Santa Cruz, CA, USA). Phospho-Akt (Ser473) Antibody #9271 Cell signaling (Beverly, MA, USA), p38α, sc-535 C20, (Santa Cruz, CA, USA), IKKβ #2684, Cell signaling (Beverly, MA, USA) [14].

Statistical analysis: All data are presented as the mean ± standard error of the mean of three independent experiments. SPSS version 25 software (IBM Corp., New York, US) was used to analyze data. Data were analyzed using a one-way analysis of variance, followed by a Bonferroni correction. *p*-values of <0.05 were considered to indicate a statistically significant difference [13,14].

## 3. Results

### 3.1. Active Compounds of Kaempferia galanga

**Figure 1:**
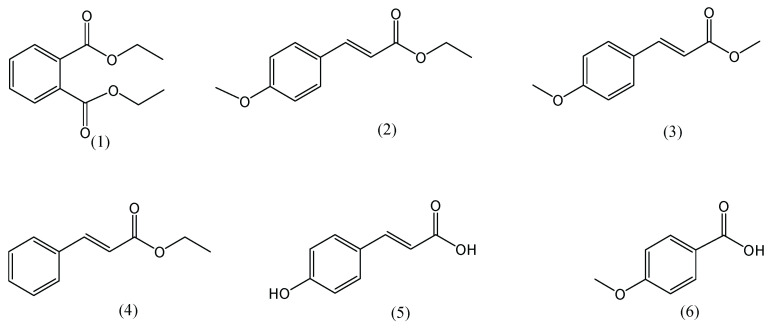
Chemical structure of the isolated compounds from *Kaempferia galanga*.

Dried rhizome of *K. galanga* was extracted with 70% EtOH to produce the ethanolic extract, and the compounds were isolated using a combination of column and preparative chromatography techniques, with silica gel as a stationary phase and various eluent systems. Six pure compounds were obtained. The compound’s structure was identified using NMR and MS spectrometry and by comparing the existing reference data for the compound [18,19,20,21]. The structure of the compound was obtained as shown in Figure 1. The compounds are diethyl-phthalate (**1**) with H-NMR (500 MHz, CDCl_3_) *δ* 7.70 (2H, m), 7.53 (2H, m), 4.21 (4H, m), 1.28 (6H, m), MS: *m/z* 222; ethyl-p-methoxycinnamate (**2**) H-NMR (500 MHz, CDCl_3_) 7.64 (1H, d,16 Hz), 7.46 (2H, d, 8 Hz), 6.88 (2H, d, 8 Hz), 6.30 (1H, d, 16 Hz), 4.25 (2H, q, 7.5 Hz), 3.81 (3H, s), 1.32 (3H, t, 8 Hz), MS: *m/z* 206; methyl-p-methoxycinnamate (**3**), H-NMR (500 MHz, CDCl_3_) 7.64 (1H, d, 16 Hz), 7.47 (2H, d, 8.5 Hz), 6.90 (2H, d, 8.5 Hz), 6.30 (1H, d, 16 Hz), 3.83 (3H, s), 3.79 (3H, s), MS: *m/z* 192; ethyl cinnamate (**4**), H-NMR (500 MHz, CDCl_3_) 7.69 (1H, d, 16 Hz), 7.51 (2H, m), 7.38 (3H, m), 6.43 (1H, d, 16 Hz), 4.27 (2H, m), 1.34 (3H, m), MS: *m/z* 176; p-hydroxycinnamic acid (**5**), H-NMR (500 MHz, CDCl_3_) 7.50 (1H, d, 16 Hz), 7.47 (2H, d, 16 Hz), 6.78 (2H, d, 16 Hz), 6.27 (1H, 8 Hz), MS: *m/z* 164; and p-methoxybenzoic acid (**6**) H-NMR (500 MHz, CD_3_OD) 7.96 (2H, d, 8 Hz), 6.97 (2H, d, 8 Hz), 3.82 (3H, s), MS: *m/z* 152.

### 3.2. NFκB Inhibition and Viability Cell against B16F10 Melanoma Cell

**Figure 2:**
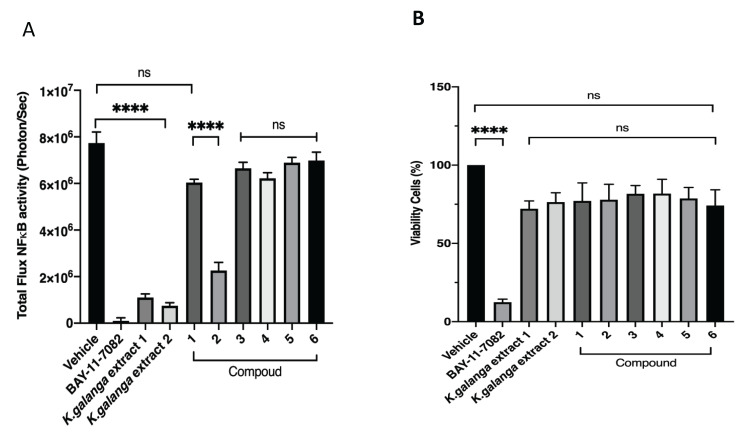
(**A**) Inhibitory activity regarding NFκB and (**B**) cell viability of B16F10 NFκB luc for isolated compounds. B16F10-NFκB luc cells were cultured for 24 h, in 10 μg/mL and 10 μM extracts and compounds, respectively, with the positive control BAY-11-7082. The test was repeated at least three times, unless explicitly stated otherwise. NFκB activity and cell viability are expressed as the percentage of viability observed in untreated cells (Bonferroni post hoc test; **** *p* < 0.0001; ns, nonsignificant).

As part of the process of exploring new bioactive components from *K. galanga*, the six isolates were then tested for their effects on NFκB gene activity and cell viability for B16F10 melanoma skin cancer cells, using the luciferin/luciferase reporter assay/WST-8 method. An investigation into the activity of nuclear factor-B (NF-B) and the viability of B16F10 NF-B-luc cells was carried out, as shown in Figure 2A,B. BAY-11-7082 was applied as a positive control.

### 3.3. Western Blotting Analysis

**Figure 3:**
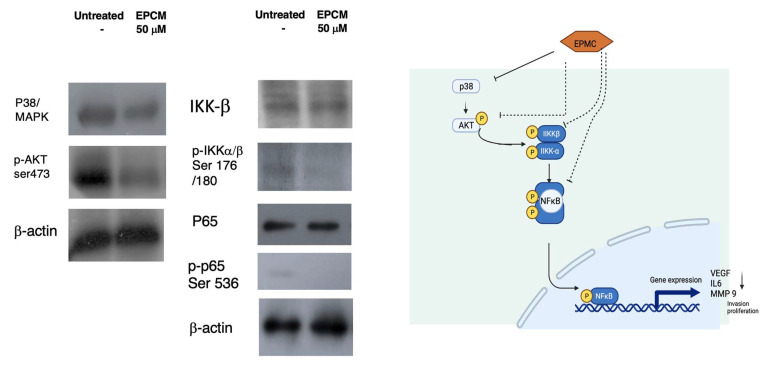
B16F10 G5-luc cells were cultured for 12 h and treated with EPMC before harvesting the protein. Furthermore, the molecular mechanism of EPMC 50 μM treatment’s effect on B16F10 G5-Luc cells was investigated by Western blotting. The original pictures of western-blot were added at Supplementary Material (Figure S1).

The Western blotting analysis result showed that EPMC 50 μM downregulated P38/MAPK, and p-Akt interacts with Akt via phospho serine 473. We hypothesize that Akt kinase interaction is required for Akt-mediated NFκB activation. Additionally, IKK-β and p-P65’s serine536 level decreased (Figure 3).

### 3.4. Migration Assay

**Figure 4:**
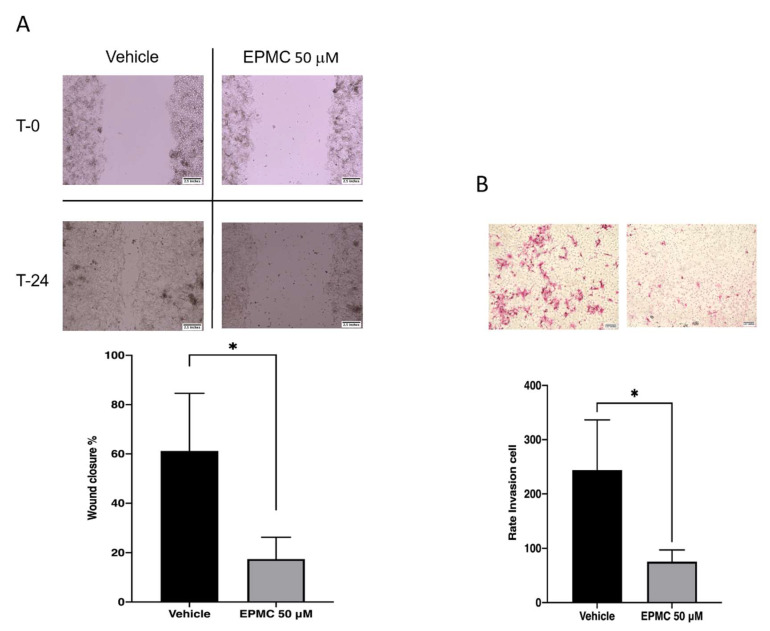
Migration assay of B16F10 G5-Luc in the presence of EPMC evaluated by the wound-healing method (**A**) and invasion using transwell. The transwell cell migration assay measures the chemotactic ability of cells toward a chemo-attractant (**B**). Wound closure and invasion cells compared with untreated cells (Bonferroni post hoc test; * *p* < 0.05).

To better understand EPMC’s possible effect on cancer activity, whether migratory or invasive, we performed a migration assay over 24 h. A concentration of 50 μM decreased melanoma cell migration into the clear area. The same result was found in the invasion assay, while a 50 μM administration of EPMC was observed in B16F10G5-Luc cells (Figure 4A,B).

### 3.5. Sensitivity Test

**Figure 5:**
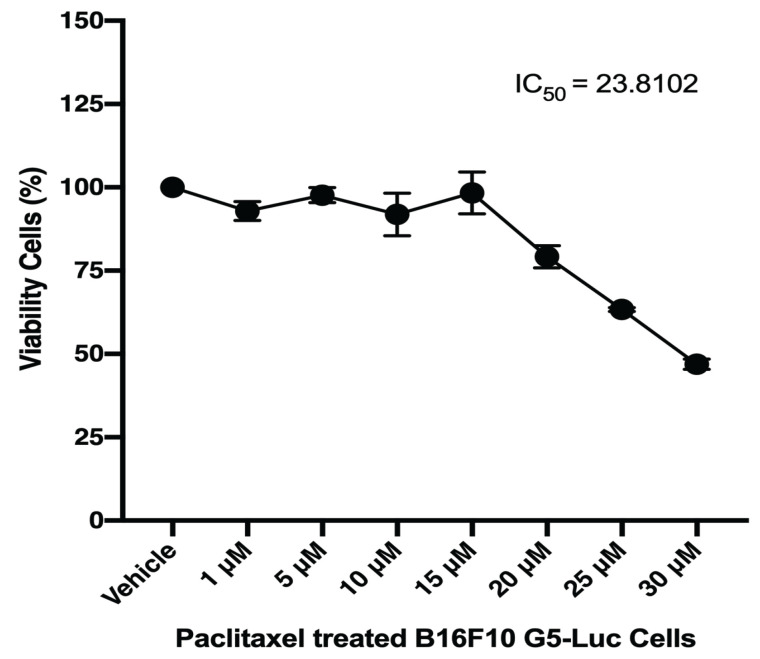
Paclitaxel-treated effect on B16F10 G5-Luc normalized by percentage of viability observed in untreated cells (Bonferroni post hoc test).

**Figure 6:**
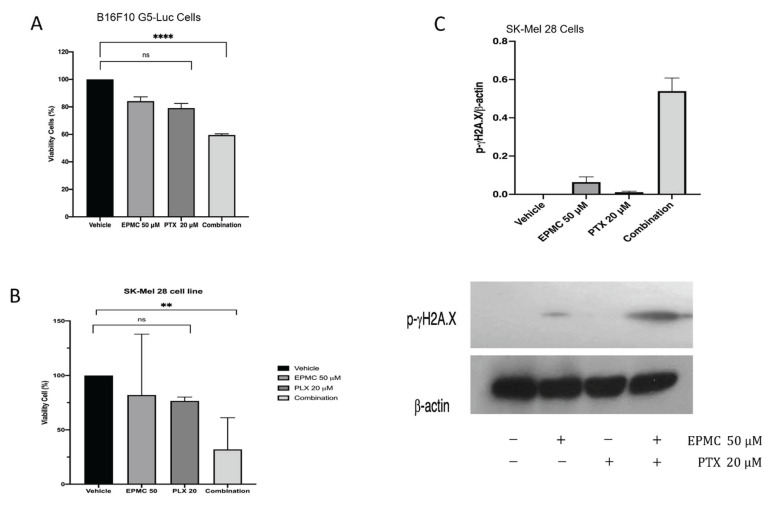
Paclitaxel and EPMC chemo-sensitizing effect on B16F10 G5-Luc (**A**) and human SK-Mel 28 melanoma cell line (**B**); cell viability normalized by percentage of viability observed in untreated cells, SK-Mel 28 cells were cultured for 12 h, then the protein was harvested. Additionally, the molecular mechanisms of EPMC 50 μM and PTX 20 μM cotreatment were analyzed using western blotting (**C**). The original pictures of western-blot were added at Supplementary Material (Figure S1). Statistical analysis by Bonferroni post hoc test; ** *p* < 0.01, **** *p* < 0.0001.

When testing sensitized paclitaxel (PTX) 1–30 μM on B16F10 G5-Luc, PTX remained resistant, with IC50 = 23.81 μM. (Figure 5). Furthermore, both compounds were used for treatment at a nontoxic dose, EMPC 50 μM and PTX 20 μM, together with the co-treatment with both EPMC and PTX of B16F10 G5-Luc and human SK-Mel 28 melanoma cancer cells. The combination showed a toxic effect compared to single treatment (Figure 6A,B). The Western blotting results indicated that SK-Mel 28 has an effect on EPMC and, combined with PTX, could activate the phosphorylation of γ-H2AX, causing a double-stranded DNA break in the cell and resulting in apoptosis induction (Figure 6C).

## 4. Discussion

Natural medicines have a significant impact on drug discovery. Some herb-derived compounds have been studied for their pharmacological activity, and some have even been used as drugs. The main compounds of *Kaempferia galanga* were analyzed in this study. The major compound was crystallized from the ethanolic extract at room temperature. Pure crystals were obtained after further purification via chromatography and recrystallization. The extract was further isolated, finding five other compounds, identified in the results.

NFκB’s gene activity and cell viability on B16F10 melanoma skin cancer cells were investigated using the luciferin/luciferase reporter assay/WST-8 method. This method uses the principle of NAD(P)H reduction of tetrazolium salt into formazan products to determine cell metabolism activity as an indicator of cell viability [22]. The NFκB gene is essential for the transcription of the NFκB protein, also known as the transcription factor protein. NFκB regulates the translocation of these proteins into the nucleus, which then activates promoter genes to carry out the transcription of pro-inflammatory and cell survival genes [23,24]. This gene is typically manipulated in abnormal cells, such as cancer cells, to allow cells to continue activating this primary regulatory gene, which in turn allows cells to survive, multiply, and stop producing the pro-inflammatory proteins that are used to inhibit cancer cell growth [25].

In this research, activity compounds from *K. galanga*, of NFκB and the viability of B16F10 NFκB luc cells were identified, as shown in Figure 2. BAY-11-7082 was used as a positive control for NFκB inhibition, even though it continued to be cytotoxic to the cancer cell line that was used [26]. The results of this study indicated that compound **2** (ethyl-p-methoxycinnamate, EPMC) inhibited cells that were transfected with the NFκB luc gene in B16F10 cells (B16F10-NFκB-Luc2). However, the other compounds demonstrated relatively similar results, with weak inhibition activity. Cytotoxicity testing on these cells revealed that none of the obtained compounds were toxic. This demonstrates that EPMC inhibits the activity of the NFκB gene and is not toxic (Figure 2A,B).

**Figure 7:**
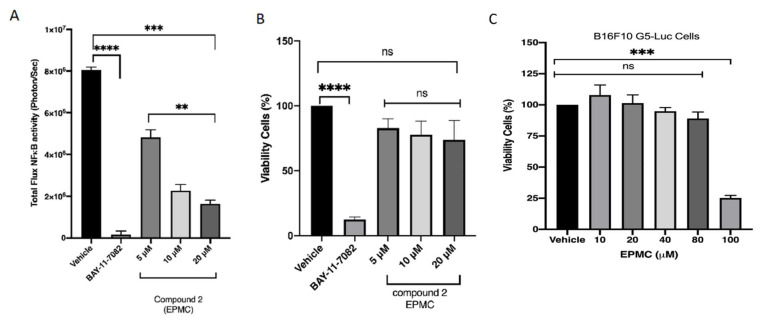
B16F10G5-luc cells were cultured for 24 h. BAY-11-7082 was used as a positive control. The test was repeated at least three times, unless explicitly stated otherwise. Inhibitory activity of NFκB (**A**) and cell viability for B16F10 NFκB luc (**B**) of 5, 10 and 20 µM and (**C**) dose response IC50 of EPMC was calculated using a calculator tool [27]. Cell viability is expressed as percentage of viability observed in untreated cells (Bonferroni post hoc test; ** *p* < 0.01, *** *p* < 0.001, **** *p* < 0.0001).

Similarly, the objective of this research was to identify compounds that, when used clinically, would not harm normal human cells but would kill cancer cells that rely on the NFκB gene for survival. An additional investigation of the EPMC at concentrations of 5, 10, and 20 μM revealed a concentration-dependent activity and established that the compound is not cytotoxic at these concentrations (Figure 7A,B). These data indicate that EPMC (**2**) is highly effective at inhibiting the NFκB gene’s activity, but it does not cause cell death at the tested concentrations (5, 10 and 20 μM). Additionally, the ensuing tests were conducted to determine the compound’s safety level using a parental cell transfected with luciferin (B16F10G5Luc cells).

As can be seen, EPMC is cytotoxic at concentrations greater than 100 μM, with an IC_50_ of 88.7 μM (Figure 7C). As a result, further investigation with a nontoxic dose and a high level of NFκB inhibition is necessary. This research was able to inhibit the NFκB regulatory gene’s activity in these melanoma cancer cells. NFκB has been shown to evoke invasion and metastasis and pro-inflammatory genes in cancerous cells, making them more resistant to therapy. Hence, inhibiting NFκB has long been considered an effective strategy for delaying the onset of cancer [28]. To assess NFκB’s role as a transcription factor, we examined the mechanism of action of this targeted therapy. Additionally, we discovered that the downregulation of NFκB is regulated by their upstream proteins p38/ERK and AKT. The changes in NFκB play a critical role on phosphorylation of serine536 and NFκB’s transcriptional activity. EPMC treatment could dephosphorylate the p65 NFκB sub-unit and inhibit transcriptional activity. The research showed that EPMC mostly interacts on phosphorylation Akt serine473. It also hypothesized that Akt kinase interaction is required for the Akt-mediated enhancement of NFκB activity, and that this process is related to the level of inhibition of p38/MAPK. This is intimately connected to the signaling pathways p38MAPK/Akt/NFκB (Figure 3). However, further investigation of EPMC regulation in membrane-bound receptors is required.

Furthermore, while gaining a better understanding of the possible effect of EPMC on cancer cells’ ability to migrate or invade in relation to their ability to regulate the P38/AKT/NFκB pathway, it was found that EPMC administration also had an effect on B16F10G5-Luc cells’ ability to migrate or invade (Figure 4A,B). The Boyden Chamber system was utilized in the invasion assay. The movement of a cell in was tested in response to a chemical stimulus. Simple staining was used to investigate cell movement, with the morphology of the cells increasing as they move. Cells in melanoma usually acquire invasive abilities through the process known as epithelial-to-mesenchymal transition (EMT) [29]. A similar migratory pattern was observed in B16F10G5-Luc cells in both wound healing and Boyden chamber migratory assays, suggesting that they may be related.

A new way to beat cancer chemoresistance with natural compounds could be by using the B16F10-NFκB-Luc cell line [30]. EPMC was identified to be an effective inhibitor of NFκB activation in B16F10-NFκB-Luc cells, while paclitaxel (PTX) reportedly induced NFκB activation, which affected resistance to cancer cell treatment [31]. To determine EPMC’s ability to sensitize PTX, both compounds were co-treated in a non-toxic dose. The result indicated that PTX maintains resistance with IC50 23.81 μM on B16F10 G5-Luc melanoma cancer cells (Figure 5). Interestingly, the addition of EPMC (50 μM) to PTX (20 μM) activates the PLX’s sensitivity to kill B16F10 cancer cells (Figure 6A). Thus, it will be necessary to determine whether the sensitivity of the two compounds is due to the reduction in these cells’ ability to survive, because of the NFκB gene being inhibited by the NFκB inhibitor compound that was discovered in EPMC. The activity was continued for paclitaxel in combination with EPMC 50 μM using human malignant melanoma cancer cells, SK-Mel 28 a V600E type BRAF Mutation (Figure 6B). The molecular mechanism, as determined by protein expression, indicates that this is due to EPMC’s ability to activate the phosphorylation of γ-H2AX, which means that the co-culture of EPMC and PTX resulted in a double-stranded DNA break in the cell, resulting in cell viability reduction (Figure 6C). EPMC significantly enhances the toxicity of PLX against the SK-Mel 28 cell line. Interestingly, our findings might show the phosphorylation of Akt residues, Ser473 in response to DNA damage. As this has been reported to be necessary for the full activation of Akt [31], we believe that Akt signaling may play an important role in the regulation of γH2A.X phosphorylation as well [32,33]. As it was identified that the two compounds’ sensitivity is due to an increase in phosporilation-γH2A.X expression, a molecular marker for DNA damage might be caused by NFκB regulation. This demonstrates EPMC’s potential for clinical trials with human melanoma cancer cells. Some natural products have been reported for the inhibitory activity on melanoma cancer cell line: resveratrol, with an IC50 value range of 120–257 μM, and saikosaponin B, from *Bupleurum falcatnum*, which showed inhibitory activity with an IC50 value of 200 μM [30,34]. However, when comparing the inhibitory activity, EPMC performed more strongly than the previous reported natural products. EPMC was reported as the major compound from *K. galanga*. Other studies have reported the methanolic extract of this plant was 78% EPMC [35]. High concentrations of EPMC from this plant provide natural resources for developing a new drug and its potential use as medicinal plant.

## 5. Conclusions

The identification of a natural anti-inflammatory compound by inhibiting NFκB activity in *K. galanga* extracts represents a promising compound; ethyl p-methoxycinnamate (EPMC) is a potential candidate for the implementation of anti-melanoma agents that target the p38/AKT/NFκB pathway. Additionally, EPMC increased paclitaxel sensitivity by increasing DNA damage according to the double-stranded break marker γH2A.X. Thus, EPMC is highly effective as a natural anti-metastasis and chemosensitizer agent in vitro, allowing for further investigation into its potential in human dermatology diseases, both in vivo and clinically.

## Data Availability

Not applicable.

## Supplementary Materials

The following supporting information can be downloaded at https://www.mdpi.com/article/10.3390/life12030337/s1. Figure S1: original Western blot image.

## References

[1] Chin Y.W., Balunas M.J., Chai H.B., Kinghorn A.D. (2006). Drug discovery from natural sources. AAPS J..

[2] Jirovetz L., Buchbauer G., Shafi P.M., Abraham G.T. (2001). Analysis of the essential oil of the roots of the medicinal plant *Kaempferia galanga* L. (Zingiberaceae) from South-India. Act. Pharm. Turc..

[3] Lallo S., Hasmiranti A., Hardianti B. (2020). Effect of the growth environment on Patchouli (*Pogostemon cablin* Benth.) oil character at Southeast Sulawesi, Indonesia. Med. Plants Int. J. Phytomed. Relat. Ind..

[4] Khairullah A.R., Solikhah T.I., Ansori A.N.M., Hanisia R.H., Puspitarani G.A., Fadholly A., Ramandinianto S.C. (2021). Medicinal importance of *Kaempferia galanga* L. (Zingiberaceae): A comprehensive review. J. Herbmed Pharmacol..

[5] Elshamy A.I., Mohamed T.A., Essa A.F., Gawad A.-E., Ahmed M., Alqahtani A.S., Shahat A.A., Yoneyama T., Farrag A.R.H., Noji M. (2019). Recent advances in Kaempferia phytochemistry and biological activity: A comprehensive review. Nutrients.

[6] Srivastava N., Singh S., Gupta A.C., Shanker K., Bawankule D.U., Luqman S. (2019). Aromatic ginger (*Kaempferia galanga* L.) extracts with ameliorative and protective potential as a functional food, beyond its flavor and nutritional benefits. Toxicol. Rep..

[7] Jagadish P.C., Latha K.P., Mudgal J., Nampurath G.K. (2016). Extraction, characterization and evaluation of *Kaempferia galanga* L. (*Zingiberaceae*) rhizome extracts against acute and chronic inflammation in rats. J. Ethnopharmacol..

[8] He Z.-H., Yue G.G.-L., Lau C.B.-S., Ge W., But P.P.-H. (2012). Antiangiogenic effects and mechanisms of trans-ethyl p-Methoxycinnamate from *Kaempferia galanga* L.. J. Agric. Food Chem..

[9] Amuamuta A., Plengsuriyakarn T., Na-Bangchang K. (2017). Anticholangiocarcinoma activity and toxicity of the *Kaempferia galanga* Linn. rhizome ethanolic extract. BMC Complement. Altern. Med..

[10] Kumar A. (2020). Phytochemistry, pharmacological activities and uses of traditional medicinal plant *Kaempferia galanga* L.—An overview. J. Ethnopharmacol..

[11] Shetu H.J., Trisha K.T., Sikta S.A., Anwar R., Rashed S.S.B., Dash P.R. (2018). Pharmacological importance of *Kaempferia galanga* (Zingiberaceae): A mini review. Int. J. Res. Pharm. Pharm. Sci..

[12] Xia Y., Shen S., Verma I.M. (2014). NF-κB, an active player in human cancers. Cancer Immunol. Res..

[13] Lallo S., Hardianti B., Sartini S., Ismail I., Hayakawa Y. (2022). Anti-inflamatory and cytoprotective effect of *Kaempferia galanga* extracts by targeting NFκB activity. Asian J. Plant Sci..

[14] Takahashi K., Takeda K., Saiki I., Irimura T., Hayakawa Y. (2013). Functional roles of tumor necrosis factor-related apoptosis-inducing ligand-DR5 interaction in B16F10 cells by activating the nuclear factor-κB pathway to induce metastatic potential. Cancer Sci..

[15] Yarrow J.C., Perlman Z.E., Westwood N.J., Mitchison T.J. (2004). A high-throughput cell migration assay using scratch wound healing, a comparison of image-based readout methods. BMC Biotechnol..

[16] Jonkman J.E., Cathcart J.A., Xu F., Bartolini M.E., Amon J.E., Stevens K.M., Colarusso P. (2014). An introduction to the wound healing assay using live-cell microscopy. Cell Adh. Migr..

[17] Shin M.K., Sasaki F., Ki D.W., Win N.N., Morita H., Hayakawa Y. (2021). Anti-metastatic effects of ergosterol peroxide from the entomophatogenic fungus *Ophiocordyceps gracilioides* on 4T1 breast cancer cells. J. Nat. Med..

[18] Keire D.A., Anto P., Faull K.F., Ruth E., Walsh J.H., Chew P., Quisimoro D., Territo M., Reeve J.R. (2001). Diethyl phatalate, a chemotactic factor secreted by Helicobacter pylori. J. Biol. Chem..

[19] Hasali N.H.M., Umar N.M., Zuberdi A.M., Alfarra H.Y. (2013). Biotransformation of ethyl p-methoxycinnamate from *Kaempferia galanga* L. using *Aspergillus niger*. Int. J. Biosci..

[20] Swislocka R., Kwoczuk-Sadowi M., Kalinowska M., Lewandowski W. (2012). Spectroscopic (FT-IR, FT-Raman, 1H and 13C NMR) and theoretical studies of p-coumaric acid and alkali metal p-coumarates. Spectroscopy.

[21] Hasegawa T., Hashimoto M., Fujihara T., Yamada H. (2016). Aroma profile of galangal composed of cinnamic acid derivatives and their structure-odor relationships. Nat. Prod. Commun..

[22] Präbst K., Engelhardt H., Ringgeler S., Hübner H. (2017). Basic colorimetric proliferation assays: MTT, WST, and resazurin. Cell Viability Assays.

[23] Martincuks A., Andryka K., Küster A., Schmitz-Van de Leur H., Komorowski M., Müller-Newen G. (2017). Nuclear translocation of STAT3 and NF-κB are independent of each other but NF-κB supports expression and activation of STAT3. Cell. Signal..

[24] Bhatt D., Ghosh S. (2014). Regulation of the NF-κB-mediated transcription of inflammatory genes. Front. Immunol..

[25] Li F., Zhang J., Arfuso F., Chinnathambi A., Zayed M., Alharbi S.A., Kumar A.P., Ahn K.S., Sethi G. (2015). NF-κB in cancer therapy. Arch. Toxicol..

[26] Rauert-Wunderlich H., Siegmund D., Maier E., Giner T., Bargou R.C., Wajant H., Stühmer T. (2013). The IKK inhibitor Bay 11-7082 induces cell death independent from inhibition of activation of NFκB transcription factors. PLoS ONE.

[27] Quest Graph™ IC50 Calculator AAT Bioquest, Inc. https://www.aatbio.com/tools/ic50-calculator.

[28] Dolcet X., Llobet D., Pallares J., Matias-Guiu X. (2005). NF-κB in development and progression of human cancer. Virchows Arch..

[29] Guy J.B., Espenel S., Vallard A., Batiston-Montagne P., Wozny A.S., Ardail D., Alphonse G., Rancoule C., Rodriguez-Lafrasse C., Magne N. (2017). Evaluation of the cell invasion and migration process: A comparison of the video microscope-based scratch wound assay and the boyden chamber assay. J. Viz. Exp..

[30] Ma H., Yokoyama S., Saiki I., Hayakawa Y. (2017). Chemosensitizing effect of saikosaponin B on B16F10 melanoma cells. Nutr. Cancer.

[31] An J., Huang Y.-C., Xu Q.-Z., Zhou L.-J., Shang Z.-F., Huang B., Wang Y., Liu X.-D., Wu D.-C., Zhou P.-K. (2010). DNA-PKcs plays a dominant role in the regulation of H2AX phosphorylation in response to DNA damage and cell cycle progression. BMC Mol. Biol..

[32] Guo Z.-F., Kong F.-L. (2021). Akt regulates RSK2 to alter phosphorylation level of H2A. X in breast cancer. Oncol. Lett..

[33] Brown K.K., Montaser-Kouhsari L., Beck A.H., Toker A. (2015). MERIT40 Is an Akt substrate that promotes resolution of DNA damage induced by chemotherapy. Cell Rep..

[34] Habibie H., Yokoyama S., Abdelhamed S., Awale S., Sakurai H., Hayakawa Y., Saiki I. (2014). Survivin suppression through STAT3/β-catenin is essential for resveratrol-induced melanoma apoptosis. Int. J. Oncol..

[35] Winingsih W., Husein S.G., Ramdhani R.P.N. (2021). Analysis of ethyl p-methoxycinnamate from *Kaempferia galanga* L. extract by high performance liquid chromatography. J. Trop. Pharm. Chem..

