# Supplementary material for: Ethyl P-Methoxycinnamate: An Active Anti-Metastasis Agent and Chemosensitizer Targeting NFκB from Kaempferia galanga for Melanoma Cells
Source: Life (Basel). 2022 Feb 24;12(3):337. doi: 10.3390/life12030337 (PMC8950268; doi:10.3390/life12030337)
Supplement: Supplementary file 1 [file life-12-00337-s001.zip › life-1597053-supplementary.pdf]

Ethyl P-Methoxycinnamate: An Active Anti-Metastasis Agent and Chemosensitizer Targeting NFkB from Kaempferia galanga for Melanoma Cells

Subehan Lallo [1,*], Besse Hardianti [2,3], Sartini Sartini [1], Ismail Ismail [1], Dewi Laela [1] and Yoshihiro Hayakawa [3,*]

1 Faculty of Pharmacy, Hasanuddin University, Makassar 90245, Indonesia; (sardj@farmasi.unhas.ac.id); (is-mail@unhas.ac.id); (dewiprimayanti@unhas.ac.id)
2 Sekolah Tinggi Ilmu Farmasi Makassar, Makassar, Indonesia; bessehardianti@stifa.ac.id
3 Institute of Natural Medicine, University of Toyama, Toyama, Japan
* Correspondence: subehan@unhas.ac.id (S.L.); haya@inm.u-toyama.ac.jp (Y.H.)

Supplementary Material:

Figure S1: Original of western blot image of protein expression in presence and absence of ethyl p-methoxycinnamate.

A. P38/MAPK

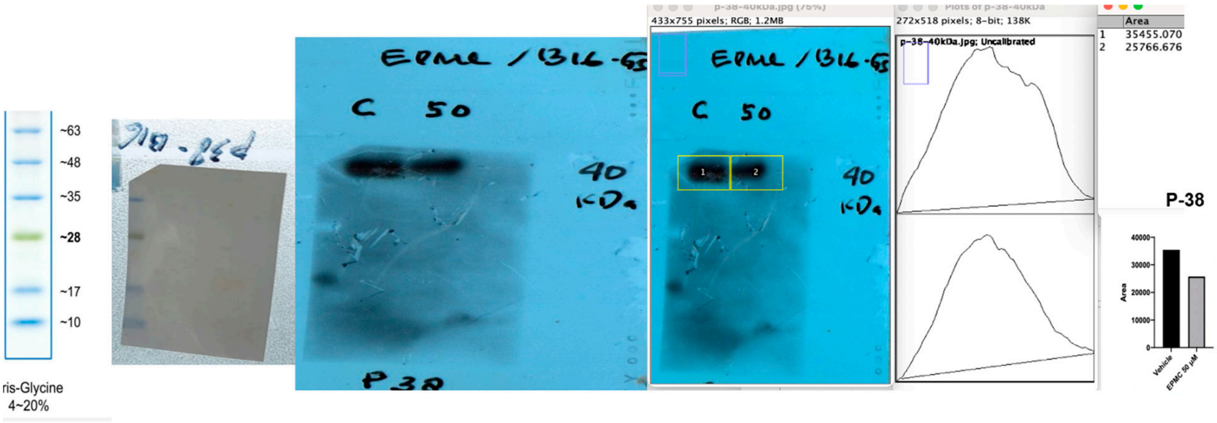

B. p-AKT ser473

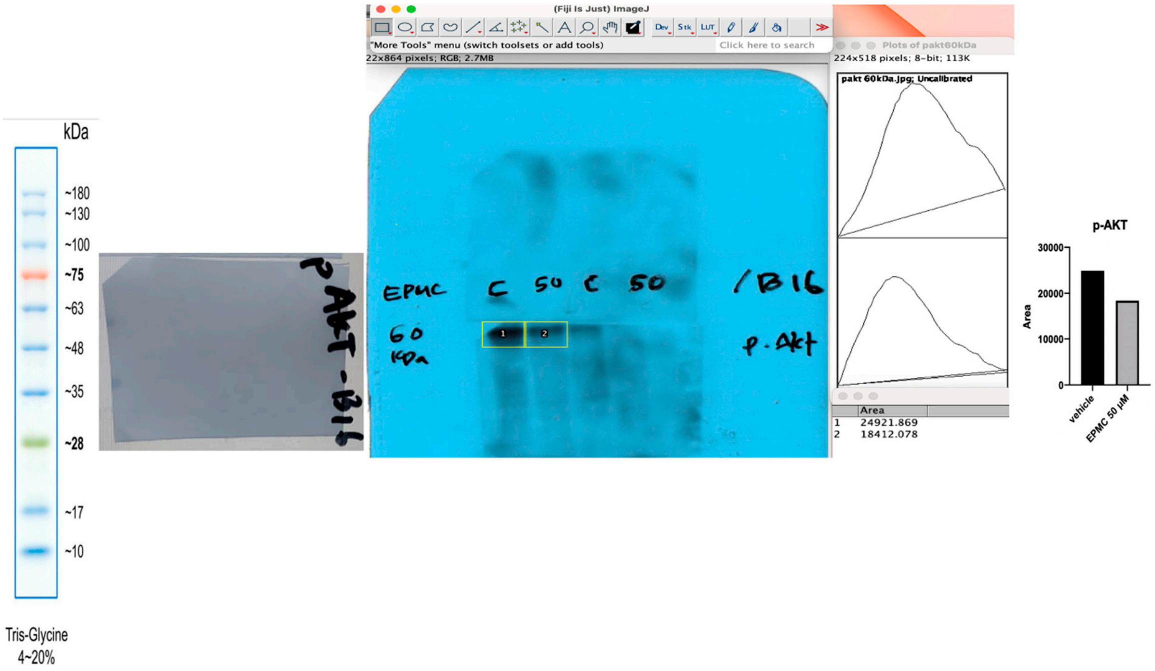

C. β-Actin

B16F10Luc-G5 Cells

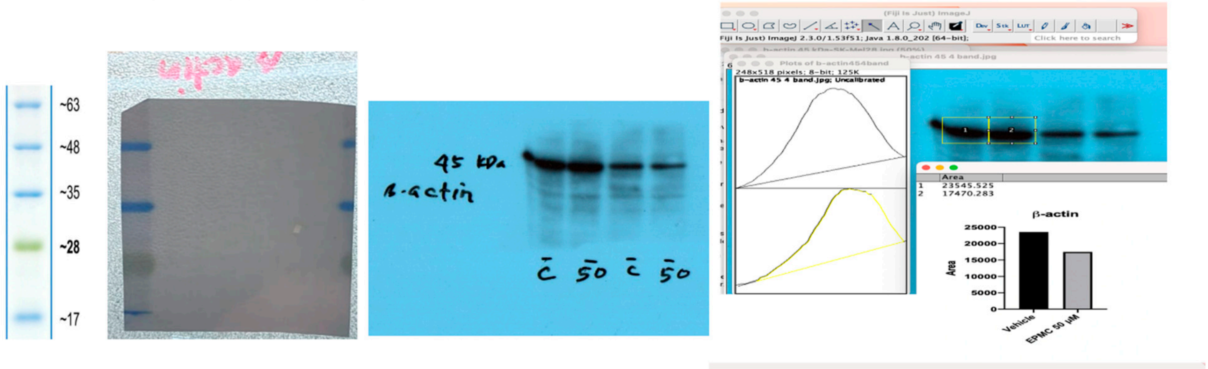

D. IKK-β

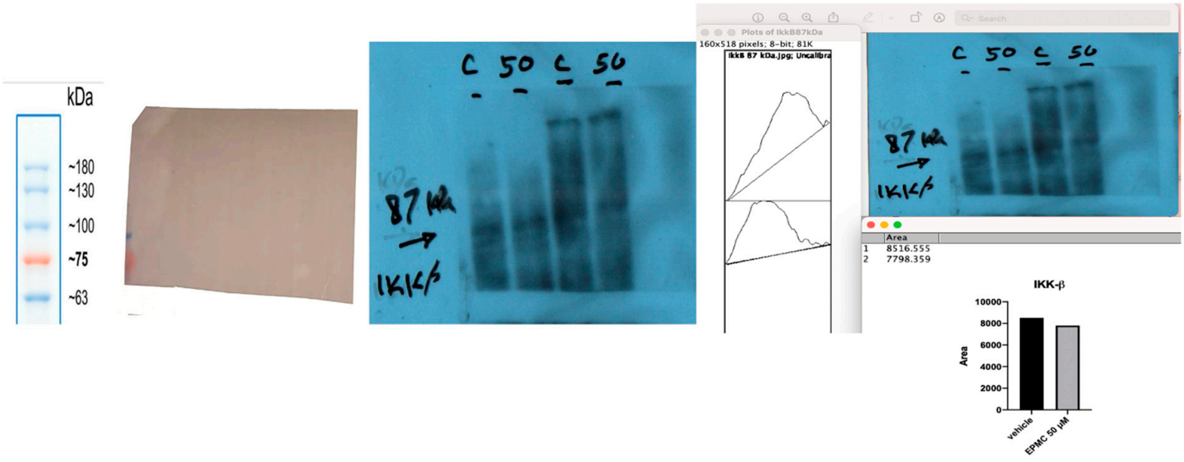

E. p-IKKα/β ser176/180

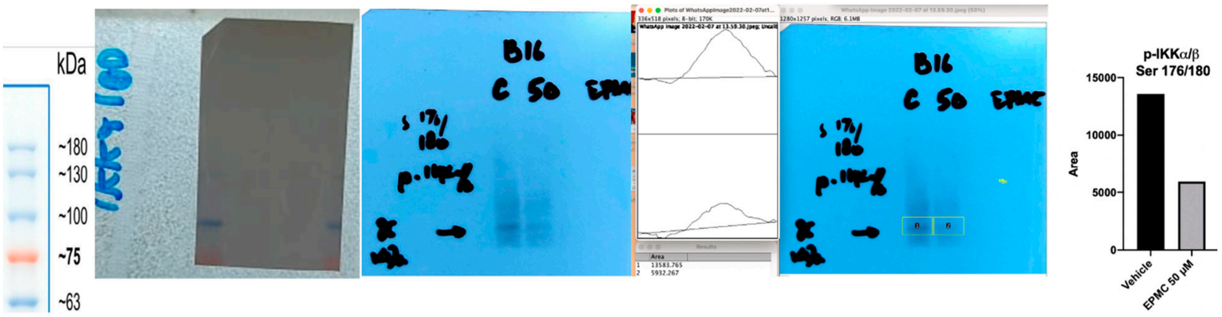

F. P65

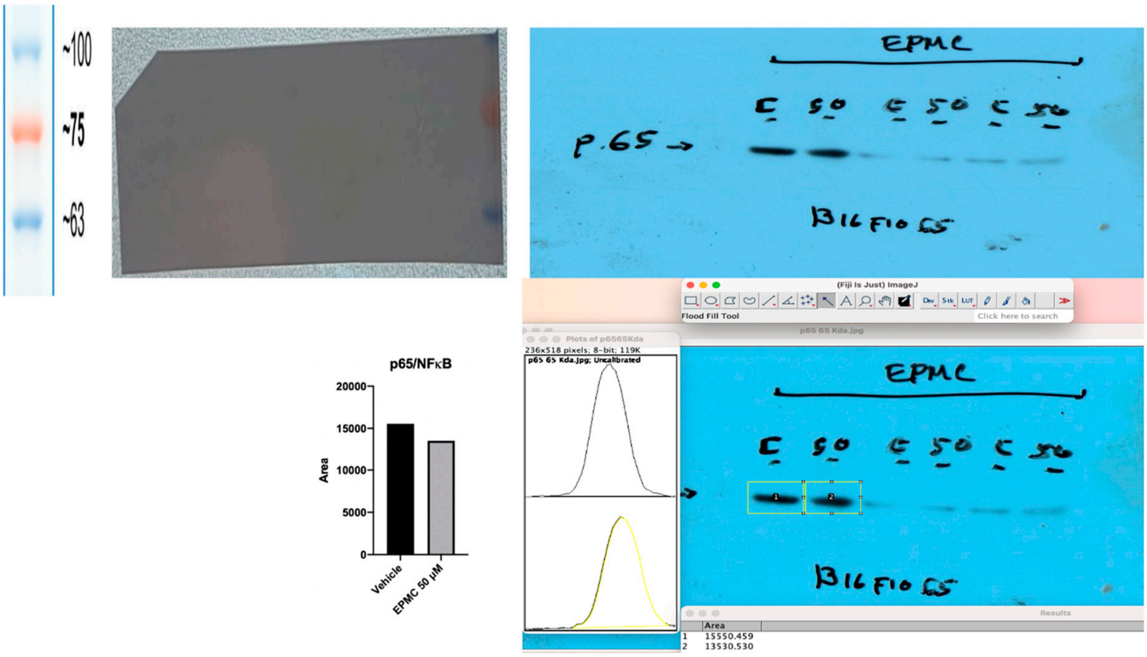
~100
~75
~63
EPMC
p65
B16F10 G5
(Fiji Is Just) ImageJ
Flood Fill Tool
Plots of p6565Kda
236x518 pixels; 8-bit; 119K
p65 65 Kda.jpg; Uncalibrated
p65/NFκB
Area
20000
15000
10000
5000
0
Vehicle
EPMC 50 μM
Results
Area
1 15550.459
2 13530.530

G. p-p65

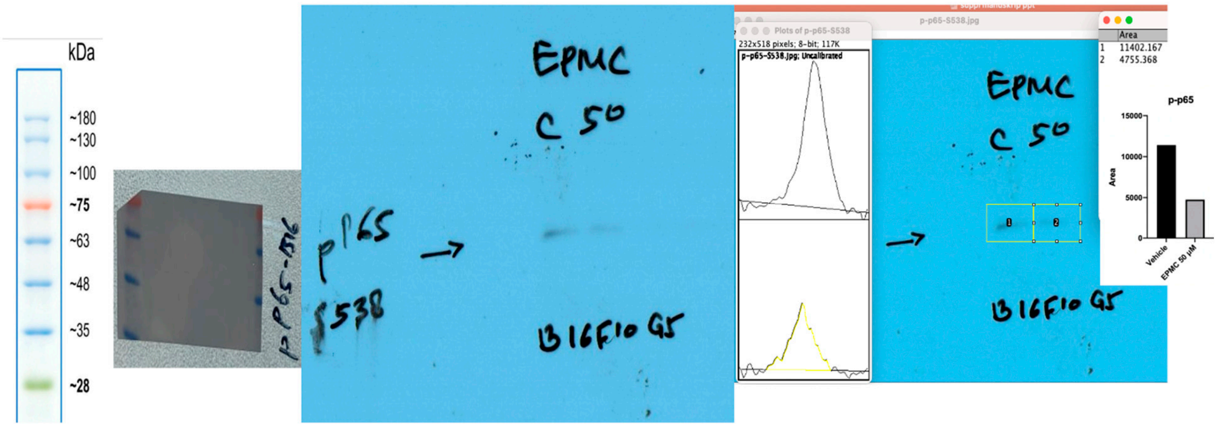
kDa
~180
~130
~100
~75
~63
~48
~35
~28
EPMC
C 50
p65
S538
B16F10 G5
Plots of p-p65-S538
232x518 pixels; 8-bit; 117K
p-p65-S538.jpg; Uncalibrated
p-p65-S538.jpg
Area
1 11402.167
2 4755.368
p-p65
15000
10000
5000
0
Area
Vehicle
EPMC 50 μM

H. β-actin

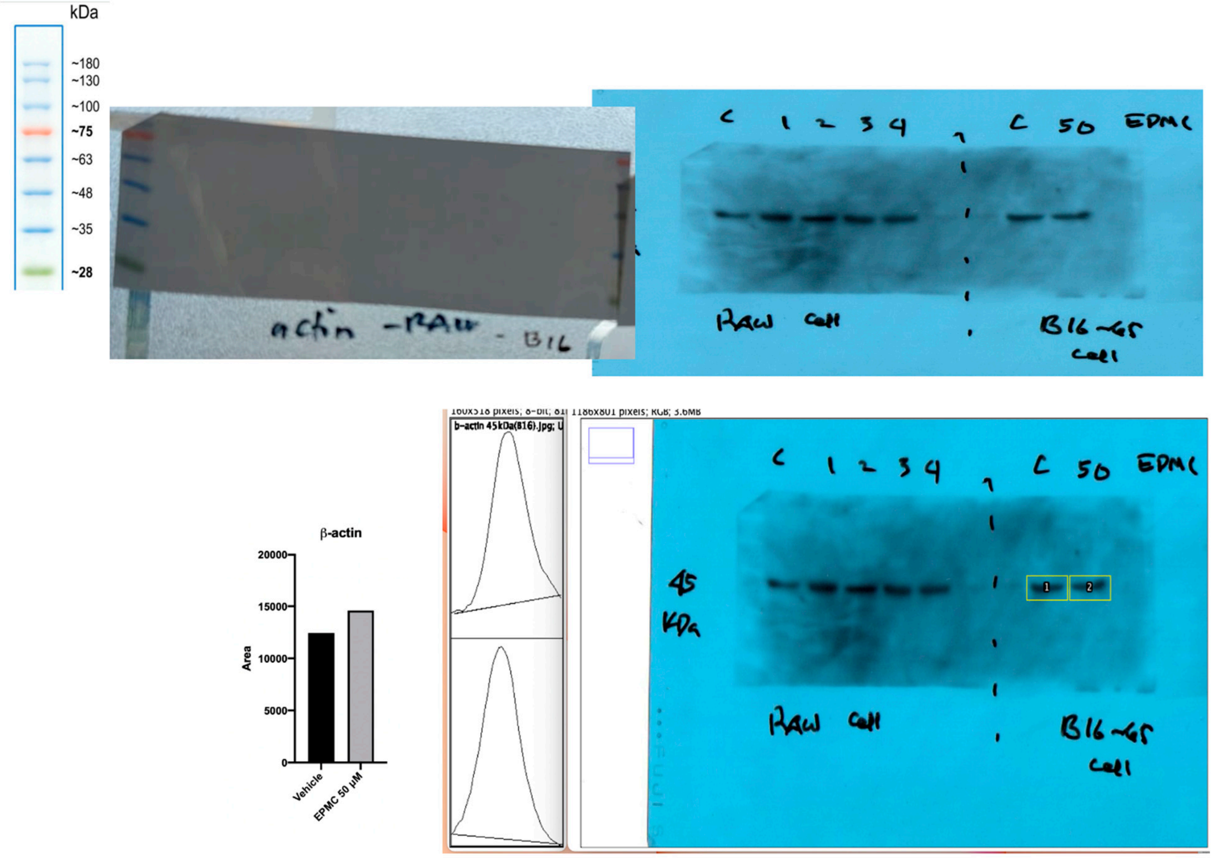

I. p-H2A.X

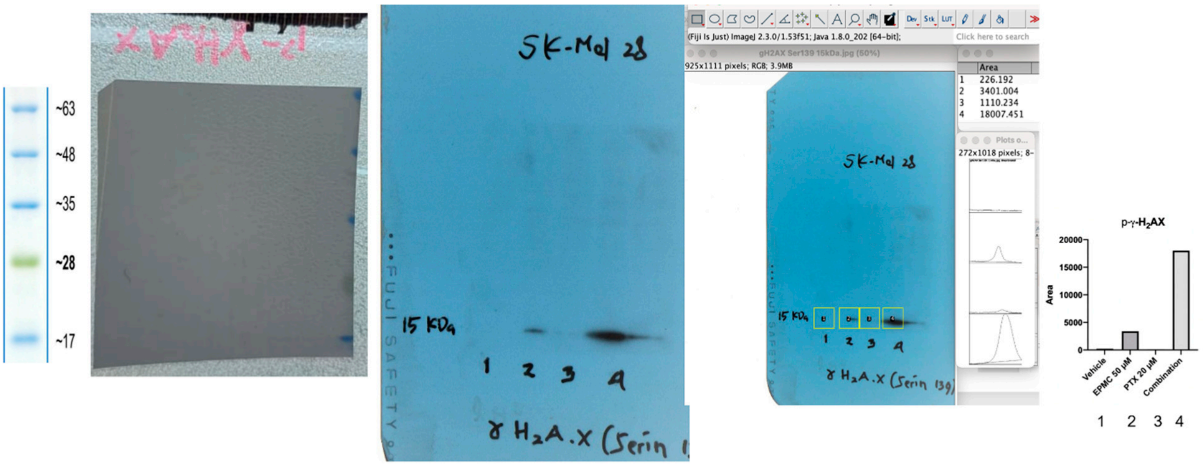

J. β-Actin

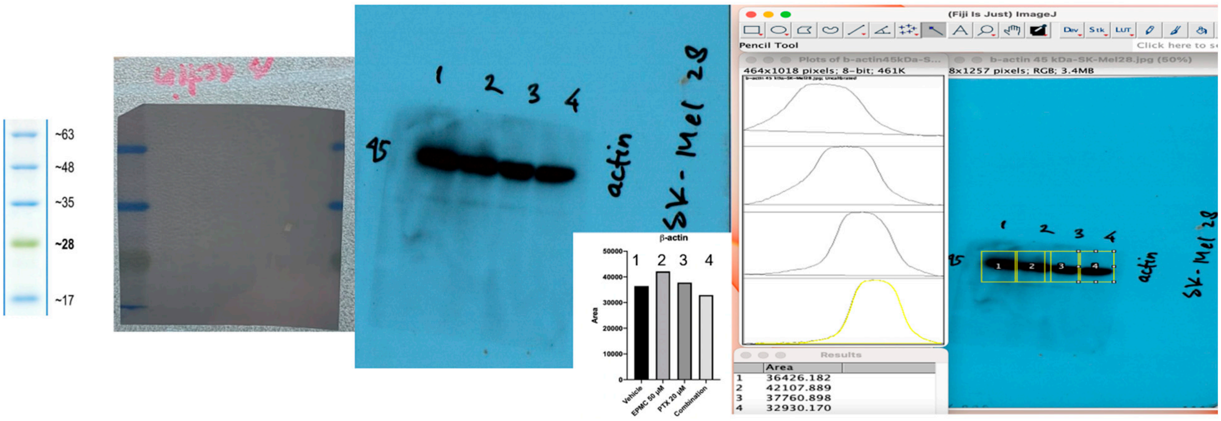

~63
~48
~35
~28
~17
1 2 3 4
45
actin
SK-Mel 28
β-actin
1 2 3 4
50000
40000
30000
20000
10000
0
Area
Vehicle
EPMC 50 μM
PTX 20 μM
Combination
(Fiji Is Just) ImageJ
Pencil Tool
464x1018 pixels; 8-bit; 461K
8x1257 pixels; RGB; 3.4MB
Results
Area
1 36426.182
2 42107.889
3 37760.898
4 32930.170
